# Patients with Epididymo-Orchitis and Meteorological Impact in Taiwan: A Nationwide Population-Based Study

**DOI:** 10.1155/2017/1506857

**Published:** 2017-02-20

**Authors:** Jui-Ming Liu, Ying-Hsu Chang, Te-Wei Ho, Fung-Wei Chang, See-Tong Pang, Ren-Jun Hsu, Po-Hung Lin

**Affiliations:** ^1^Division of Urology, Department of Surgery, Taoyuan General Hospital, Ministry of Health and Welfare, Taoyuan, Taiwan; ^2^Division of Urology, Department of Surgery, Chang Gung Memorial Hospital, Taoyuan, Taiwan; ^3^Graduate Institute of Biomedical Electronics and Bioinformatics, National Taiwan University, Taipei, Taiwan; ^4^Department of Obstetrics & Gynecology, Tri-Service General Hospital, National Defense Medical Center, Taipei, Taiwan; ^5^Graduate Institute of Life Sciences, National Defense Medical Center, Taipei, Taiwan; ^6^Biobank Management Center, Tri-Service General Hospital, National Defense Medical Center, Taipei, Taiwan; ^7^Parasitology, Tri-Service General Hospital, National Defense Medical Center, Taipei, Taiwan; ^8^Graduate Institute of Clinical Medical Sciences, College of Medicine, Chang Gung University, Taoyuan, Taiwan

## Abstract

*Background*. Epididymo-orchitis is a common infectious disease among men, especially men aged 20 to 39 years. The aim of this study was to analyze possible associations of various meteorological indicators on the incidence of epididymo-orchitis in Taiwan.* Methods and Materials*. This nationwide population-based study collected data on cases of epididymo-orchitis that were newly diagnosed from 2001 to 2013 in Taiwan. Monthly meteorological indicators, including average temperatures, humidity, rainfall, total rain days, and sunshine hours, were collected from the Central Weather Bureau of Taiwan. Data for a total of 7,233 patients with epididymo-orchitis were collected for this study.* Results*. The monthly incidence of epididymo-orchitis was positively correlated with temperature, rainfall, and sunshine hours. The average monthly temperature had a linear correlation with the incidence of epididymo-orchitis (ß = 0.11). The monthly average temperature is significantly related, with a positive linear correlation, to the incidence of epididymo-orchitis in Taiwan.* Conclusion*. This finding may constitute useful information in terms of helping physicians to distinguish between patients with epididymo-orchitis and testicular torsion in hot or cold weather.

## 1. Introduction

Epididymo-orchitis consists of inflammation of the epididymis and testes. There are nearly 600,000 cases of epididymo-orchitis per year in the United States, accounting for roughly 1/144 (0.69%) outpatient visits among 18- to 50-year-old men, with the majority of those patients aged 18 to 35 years [1]. Mittemeyer et al. conducted a study of 610 cases among soldiers in the United States army and their family members and found that most of the patients were aged 20 to 29 years. However, almost all ages were involved (with a range of 4 months to 76 years) [2]. Acute epididymitis is characterized by acute testicular pain and most commonly involves the symptom of acute scrotum as well. Orchitis usually originates from inflammation of the epididymis [3]. The symptoms of epididymo-orchitis include swelling and tenderness of the epididymis or scrotum in about 75% patients. Some patients even presented with bloodstream infections, sepsis, or septic shock due to the underlying pathogens [4].

Acute scrotum is a symptom that consists of an acute painful swelling of the scrotum. Epididymo-orchitis and testicular torsion should be considered in making a differential diagnosis when the symptom of acute scrotum occurs [5, 6]. Seasonal variations are known to occur with respect to some infectious diseases and may be due to the epidemiology of the prevalent pathogens, changes in environmental and meteorological factors, and alterations in human behavior. For example, seasonal fluctuations in urinary tract infections have been reported in several studies [7, 8]. The present study sought to evaluate the effect of various meteorological indicators on the incidence of epididymo-orchitis. More specifically, we conducted a 14-year population-based study to evaluate the relationship between meteorological indicators and epididymo-orchitis in Taiwan.

## 2. Methods

### 2.1. Data Source

This study is a nationwide population-based investigation that utilized data from Taiwan's National Health Insurance Research Database (NHIRD). The data in the NHIRD comes from the National Health Insurance (NHI) program, which began in 1995 and covered 99.9% of Taiwan's 23 million residents as of the end of 2013 [9]. All the medical claims data of inpatients and outpatients are included in the NHIRD. More specifically, this study utilized the Longitudinal Health Insurance Database 2000 (LHID2000) [10], a subdataset of the NHIRD. The LHID2000 includes data from January 2000 to December 2013 for a randomly selected sample of one million people out of the 23 million people included in the NHIRD in the year 2000. The sample of patients included in the LHID2000 has a similar demographic distribution and origin to the broader population included in the NHIRD [11]. All the clinical diagnoses in this study were made according to the International Classification of Diseases, 9th revision, Clinical Modification (ICD-9-CM).

The meteorological data utilized in this study was provided by Taiwan's Central Weather Bureau (CWB) and consists of data collected from 27 CWB weather stations distributed across various territories of Taiwan (i.e., the islands of Taiwan, Penghu, Kinmen, and Lienchiang). The monthly meteorological data included temperature (measured in degree Celsius), relative humidity (measured in percentage), total rainfall amount (measured in millimeters), total rain days (measured in days), and total sunshine hours (measured in hours) [12]. According to the weather typical of Taiwan, the months of March, April, and May constitute the spring season; June, July, and August constitute the summer; September, October, and November are the fall; and December, January, and February comprise winter.

### 2.2. Ethics Statement

This study was conducted after we received approval from the Institutional Review Board of Chang Gung Memorial Hospital at Linkou (CGMH IRB 103-2071B). As this was a retrospective study and all data was anonymous, the Institutional Review Board department agreed with the authors that it was not necessary to obtain patient consent.

### 2.3. Study Subjects

**Figure 1:**
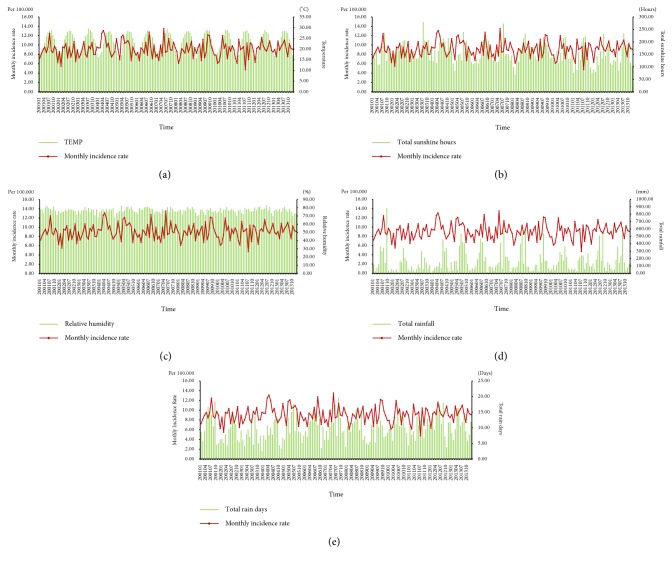
The monthly incidence of epididymo-orchitis and the corresponding monthly weather data during the study period. (a) Epididymo-orchitis incidence and monthly average temperature. (b) Epididymo-orchitis incidence and monthly total sunshine hours. (c) Epididymo-orchitis incidence and monthly relative humidity. (d) Epididymo-orchitis incidence and monthly total rain fall. (e) Epididymo-orchitis incidence and monthly total rain days.

The study subjects consisted of patients included in the LHID2000 who were newly diagnosed with epididymitis and orchitis (ICD-9-CM: 604), received a prescription for antibiotic medication, and received testicular sonography examination between January 2000 and December 2013 from the LHID2000 (Figure 1). More specifically, data for patients from administrative regions without CWB weather stations were excluded. In addition, patients who were diagnosed before December 31st, 2000, or after January 1st, 2013 (*n* = 438), and patients with incomplete demographic data (*n* = 1,645) were also excluded. Finally, data for a total of 7,223 patients with epididymo-orchitis were collected and analyzed in this study. The diagnoses of epididymo-orchitis were based on detailed clinical examinations, with typical symptoms including painful swelling of the scrotum that may radiate to the lower abdomen, fever, frequency and urgency in voiding, and dysuria. The diagnoses of epididymo-orchitis were made by urologists, infectious disease physicians, or licensed physicians. All the patients included in this study were under antibiotic treatment.

### 2.4. Statistical Analysis

Descriptive statistics for the characteristics of study subjects and meteorological data were first calculated by Student's *t*-test and Chi-square test; Spearman's rank correlation was used to examine the relationship between the meteorological factors and the monthly incidence rates of epididymo-orchitis. The linear regression model was also used to estimate the relationship between the meteorological factors and monthly incidence rates of epididymo-orchitis. All the tests were two-sided, with *p* value < 0.05 being regarded as statistically significant. All the statistical analyses were performed with SAS 9.2 software.

## 3. Results

Data for a total of 7,223 patients with epididymo-orchitis was collected and analyzed in this 14-year nationwide population-based study. The demographic characteristics of those patients are listed in Table 1. The mean age of male patients with epididymo-orchitis was 43.46 ± 20.03 years, with a major proportion being aged 20–49 years. Most of the patients lived in Northern and suburban areas of Taiwan.

The average monthly incidence rate of epididymo-orchitis was 9.09 per 100,000 population. A comparison of the average monthly epididymo-orchitis incidence rate with the monthly average meteorological factors is shown in Table 2. During the study period from 2000 to 2013, the hottest month was July, with an average temperature of 28.72 degrees Celsius, while the coolest month was January, with an average temperature of 16.90 degrees Celsius. In addition, the month with the most sunshine hours was July, with an average of 223.84 hours, while the month with the least sunshine hours was February, with an average of 125.81 hours. The relative humidity was highest in June, with an average of 79.31%, and the lowest in December, with an average of 73.73%. The highest average total rainfall was in August, with 347.25 mm, while the lowest average total rainfall was in January, with 62.23 mm. The highest average number of rain days was in June, with 13.80 days, and the lowest average number of rain days was in December, with 7.39 days. The monthly incidence of epididymo-orchitis was highest in May and lowest in February (Table 2). The monthly epididymo-orchitis incidence rate and corresponding monthly meteorological factors during the study period are shown in Figure 1.

The correlations between the meteorological factors and the incidence rate of epididymo-orchitis are listed in Table 3. The average temperature, total rainfall amount, and total sunshine hours had statistically significant correlations with the incidence of epididymo-orchitis. However, only average temperature (ß = 0.11, *P* < 0.001) was found to have a significant linear correlation with the incidence of epididymo-orchitis after regression model analysis (Table 4).

## 4. Discussion

This is the first study to investigate the relationships between different meteorological indicators and the incidence of epididymo-orchitis in Taiwan. The results of this large nationwide population-based study demonstrate that the incidence of epididymo-orchitis is positively correlated with average temperature.

In this study, average temperature (ß = 0.11, *P* < 0.001) was found to have a significant linear correlation with the incidence of epididymo-orchitis. Lyronis et al. conducted a study of acute scrotum and found that epididymo-orchitis was the most common cause of acute scrotum and that the incidence of epididymo-orchitis was higher in summer [13]. Our study also revealed that the incidence rates of epididymo-orchitis were higher in May and July. The retrograde ascent of pathogens is the usual cause of epididymo-orchitis. Sexually transmitted* Neisseria gonorrhoeae* or* Chlamydia trachomatis* pathogens are the most common causes of epididymo-orchitis in men aged 14 to 35 years, whereas* Escherichia coli* pathogens along with urinary tract infection constitute the most common cause in men younger than 14 years and older than 35 years of age [14–17].

Potential mechanisms for the positive correlation between temperature and epididymo-orchitis infection are discussed below. Increased temperatures increase people's perspiration and total body water loss [18]. In turn, the resulting phenomena of relative dehydration and more concentrated urine with less frequent voiding increase the rate of urinary tract infections. Increased temperatures also cause peripheral vasodilation and the pooling of blood in the skin, leading to a decrease in the effective blood volume. A hot environment may also cause peripheral vasodilation and pooling of blood in the skin which means that exposure to heat can cause a decrease in effective blood volume. In contrast, exposure to cold has also been found to cause changes in the vasopressin system leading to diuresis and the increased clearance of potential pathogens of the urinary tract [19]. In addition, seasonal variations in gonorrhea and Chlamydia infections in adolescents have previously been reported, including higher positive tests rates in the summer and fall [20].

Epididymo-orchitis and testicular torsion are both accompanied by acute scrotal pain or swelling of the scrotum. Differential diagnosis between these two diseases is important due to possibility of torsion resulting in testicular infarction, which constitutes a surgical emergency. The seasonality of testicular torsion has previously been documented in several studies. Chiu et al. conducted a 10-year cohort study with 1,782 testicular torsion patients that found that January had a significantly higher incidence rate of torsion. In addition, the incidence rate of testicular torsion was negatively associated with temperature [21]. Relatedly, Mabogunje reported an increased incidence of testicular torsion between November and February [22]. Several studies have also reported increased incidences of testicular torsion during low temperature periods in Japan, the United States, Greece, and Nigeria [13, 22–24]. In contrast, the current study found that the incidence of epididymo-orchitis was increased in May and July, while a positive correlation between epididymo-orchitis and temperature was also noted. These opposing seasonality characteristics of epididymo-orchitis and testicular torsion may be helpful in distinguishing the two diseases from each other.

A study conducted in the United State found that nearly 60,000 male adults made visits to office-based physicians for epididymo-orchitis, accounting for 1/350 (0.29%) of all the visits in the period under consideration [25]. In another nationwide study in the United States, epididymo-orchitis accounted for 1/144 (0.69%) of outpatient visits among 18- to 50-year-old men [1]. The 9.09 per 100,000 population incidence of epididymo-orchitis found in the current study was lower than previous studies. The population resident in the area without CWB weather stations was excluded for having no meteorological data available. Some epididymo-orchitis patients among this population may be excluded also. This may affect the calculated incidence of epididymo-orchitis.

The general climate is Marine Subtropical or tropical climate in Taiwan with a relative small change of temperature between summer and winter. Ratkowsky et al. demonstrated a linear relationship between the growth rate of bacterial culture and temperature even within a small range of temperature in the laboratory [26]. Thus, temperature, even a relative small range of temperature, plays an important role in growth of bacteria.

The strength of the current study is that it utilized a longitudinal nationwide database with large number of subjects and a long follow-up period. However, the study also had several limitations that should also be discussed. First, the diagnosis of epididymo-orchitis was recognized according to the ICD-9 code. The ICD-9-CM code 604.90 is used for both epididymitis and orchitis. Thus, it is difficult to distinguish epididymitis from orchitis using the ICD-9 code. Second, data from diagnostic laboratory tests that can help confirm diagnoses of epididymitis and orchitis, such as urinalysis, urine culture, and C-reactive protein (CRP) level data, are not included in the NHIRD. Therefore, epididymo-orchitis caused by sexually transmitted diseases pathogens may be included in this study. The detailed reports of color Doppler ultrasonography are also not included. Third, the data regarding the meteorological indicators came from 27 weather stations monitored by Taiwan's CWB, but these 27 stations did not cover every territory of Taiwan. Finally, this study utilized a retrospective study design. Further prospective studies are thus warranted to investigate the relationship between weather and epididymo-orchitis.

## 5. Conclusion

In conclusion, various meteorological indicators, especially average temperature, appeared to significantly affect the incidence of epididymo-orchitis in Taiwan. A significant positive linear correlation was discovered between temperature and patients with epididymo-orchitis. This result may serve as a useful clinical clue to help physicians distinguish between patients with epididymo-orchitis and testicular torsion in hot or cold weather.

## Figures and Tables

**Table 1:** Demographic characteristics of study subjects of epididymo-orchitis from 2000 to 2013 in Taiwan.

Characteristics	*n* (%)
Number of cases	7,223
*Mean age (years) (standard deviation)*	43.46 (20.03)
*Age at diagnosis (years)*	
<20	751 (10.40)
20–29	1,281 (17.74)
30–39	1,371 (18.98)
40–49	1,240 (17.17)
50–59	922 (12.76)
60–69	657 (9.10)
≥70	1,001 (13.85)
*Insured region*	
Northern	3,811 (52.76)
Central	1,153 (15.96)
Southern	2,056 (28.46)
Eastern	203 (2.82)
*Urbanicity*	
Urban	2,623 (36.31)
Suburban	4,105 (56.84)
Rural	484 (6.70)
Missing	11 (0.15)

**Table 2:** Average epididymo-orchitis incidence rate and meteorological factors according to month.

Month	Monthly incidence rate		Temperature (°C)			Total sunshine hours	Relative humidity (%)	Total rainfall (mm)	Total rain days
	Mean	95% CI	Mean (°C)	Max (°C)	Minimum (°C)				
Jan	8.09	(7.54, 8.64)	16.90	21.90	10.60	128.48	75.95	62.23	8.29
Feb	7.36	(6.53, 8.20)	18.17	23.50	10.80	125.81	77.64	66.78	7.78
March	9.78	(8.77, 10.79)	19.79	24.30	12.80	139.23	75.42	77.97	9.50
April	9.46	(8.43, 10.50)	22.94	26.80	17.10	128.83	77.24	108.26	10.84
May	10.27	(9.23, 11.31)	25.86	28.70	20.50	160.26	77.85	213.51	12.09
June	9.07	(8.34, 9.82)	27.52	29.80	21.60	167.20	79.31	315.79	13.80
July	9.51	(8.37, 10.66)	28.72	30.80	22.50	223.84	77.14	291.61	11.28
Aug	9.58	(8.53, 10.62)	28.48	30.10	22.30	196.28	78.31	347.25	13.52
Sep	8.88	(7.96, 9.80)	27.39	30.00	21.20	171.50	77.44	283.08	11.52
Oct	9.46	(8.71, 10.20)	24.92	28.00	19.00	175.30	73.97	101.41	6.51
Nov	8.87	(8.16, 9.58)	22.06	25.60	16.90	139.00	75.03	95.65	7.87
Dec	8.73	(7.99, 9.48)	18.41	22.90	11.60	137.63	73.73	71.05	7.39

**Table 3:** Spearman's rank correlations between meteorological factors and monthly epididymo-orchitis incidence rates.

Variable	*r*
Temperature	0.28^*∗∗∗*^
Total sunshine hours	0.17^*∗*^
Relative humidity	0.08
Total rainfall	0.23^*∗∗*^
Total rain days	0.14

^*∗∗∗*^ *P* < 0.001, ^*∗∗*^*P* < 0.01, ^*∗*^*P* < 0.05.

**Table 4:** Regression model of meteorological factors and monthly epididymo-orchitis incidence rates.

Variable	ß
Temperature	0.11^*∗∗∗*^
Total sunshine hours	0.01^*∗*^
Relative humidity	0.04
Total rainfall	0.002^*∗*^
Total rain days	0.06

ß: coefficient of regression analysis.

^*∗∗∗*^ *P* < 0.001, ^*∗*^*P* < 0.05.
